# Primary prevention of gestational diabetes mellitus through nutritional factors: a systematic review

**DOI:** 10.1186/s12884-016-1205-4

**Published:** 2017-01-13

**Authors:** Mikel Donazar-Ezcurra, Cristina López-del Burgo, Maira Bes-Rastrollo

**Affiliations:** 1 Department of Preventive Medicine and Public Health, University of Navarra, C/Irunlarrea1, 31008 Pamplona, Navarra Spain; 2 IDISNA, Navarra’s Health Research Institute, Pamplona, Navarra Spain; 3 Institute for Culture and Society, University of Navarra, Pamplona, Navarra Spain; 4 CIBERobn, Instituto de Salud Carlos III, Madrid, Spain

**Keywords:** Gestational Diabetes Mellitus, Primary prevention, Nutritional factors, Diet and dietary supplements

## Abstract

**Background:**

Gestational diabetes mellitus (GDM), defined as any degree of glucose intolerance with onset during pregnancy, is increasing worldwide, mostly because obesity among women of reproductive age is continuously escalating. GDM is associated with adverse maternal and fetal outcomes. The aim of this article was to systematically review literature on the effectiveness of nutritional factors before or during pregnancy to prevent GDM.

**Methods:**

We assessed the primary prevention of GDM through nutritional factors, as diet and supplements. We searched on PubMed, Cochrane Databases and ClinicalTrials.gov from inception to June 2016. Clinical trials and adjusted prospective cohort studies were included.

**Results:**

Eight clinical trials and twenty observational studies assessing the association between dietary factors and primary prevention of GDM were included. Furthermore, six clinical trials and two observational studies related to supplements were also added. Only two nutritional interventions were found to significantly reduce the incidence of GDM, besides the supplements. However, the observational studies showed that a higher adherence to a healthier dietary pattern can prevent the incidence of GDM, especially in high risk population before getting pregnant.

**Conclusions:**

The results indicate that there may be some benefits of some nutritional factors to prevent GDM. However, better-designed studies are required to generate higher quality evidence. At the moment, no strong conclusions can be drawn with regard to the best intervention for the prevention of GDM.

**Electronic supplementary material:**

The online version of this article (doi:10.1186/s12884-016-1205-4) contains supplementary material, which is available to authorized users.

## Background

Gestational diabetes mellitus (GDM), defined as any degree of glucose intolerance with onset during pregnancy, is increasing worldwide. In fact, prevalence has increased by 10–100% in the last 20 years [1]. More mothers entering pregnancy as obese and of advanced maternal age has contributed to the escalation of GDM cases. Several risk factors are known for GDM; advanced maternal age, obesity, physical inactivity, parity, ethnicity, family history of type type 2 diabetes (T2D), history of macrosomic babies, and a previous history of GDM [2, 3].

GDM is associated with adverse maternal and fetal outcomes during pregnancy and long term health [4]. In order to prevent these adverse outcomes, such as T2D, it is imperative to understand the hormonal changes and altered glucose metabolism that are associated with the development of GDM during pregnancy [5]. GDM occurs when insulin receptors are not able to respond adequately to control blood sugar levels due to hormones produced in pregnancy, such as human placental lactogen, which impacts susceptible insulin receptors. This in turn causes inappropriately high levels of blood sugar. Due to the similarities in the underlying pathophysiology and risk factors of GDM and T2D, it is probable that the factors that are effective in the prevention of T2D may be successful in the prevention of GDM as well. These factors include dietary pattern, physical activity, a decrease in rates of obesity and gestational weight gain. It is important to identify population factors to reduce the increasing rates of GDM as it is doing for T2D. The aim of the researchers was to systematically review literature on clinical trials and prospective cohort studies focusing on the effectiveness of nutritional factors (diet and supplements) before or during pregnancy to prevent GDM.

The study protocol is available as a supplementary file in this article.

## Methods

We undertook this systematic review following a protocol in accordance with the current recommendations of PRISMA and MOOSE guidelines [6, 7].

No ethical approval is required for this research project.

### Search strategy

Articles were researched in the following databases: PubMed, Cochrane Databases and ClinicalTrials.gov from inception to June 2016. The keywords used for the search were: “gestational diabetes”, “diet” and “nutrition”. Another search was done for the keywords of “supplement” and “gestational diabetes”. The search strategy was done independently by two researchers, and both extracted the relevant studies to be included in this systematic review. The disagreement between the researchers was resolved consulting a third researcher in order to reach an accord. We restricted publications to English, French and Spanish languages. All the researchers have an advanced level in these three languages. The selection process of the articles began from reading the titles. After this initial phase, the selected papers were reviewed by reading their abstracts. At that time, these selected studies were separated for further analysis to identify relevant publications, according to the inclusion/exclusion criteria. For the selection of studies, inclusion criteria were adopted such as: clinical trials or adjusted prospective cohort studies, originals, and primary prevention of GDM through nutritional factors. Due to inherent limitations other epidemiological studies were excluded. Exclusion criteria were: presence of diabetes mellitus type 1 or 2 prior to pregnancy, presence of diseases prior to pregnancy requiring dietary treatment, evaluation of diet as a treatment for patients with GDM, only physical activity for prevention of GDM and research on animals. Additional articles were identified from the reference lists of relevant studies and reviews.

## Results and discussion

### Study selection

**Fig. 1:**
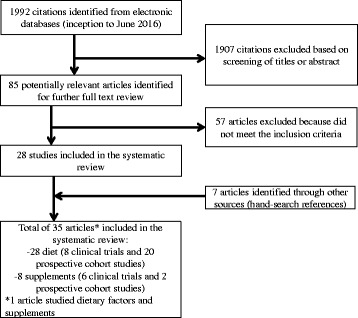
Flow-chart of prospective cohort studies and clinical trials included in the systematic review

Our initial research in the aforementioned electronic databases produced 1992 citations. Eighty-five articles were identified as potential articles for further full text review. After the next exclusion process, 28 articles were included and another seven were added from reference lists. The most important reason for exclusion was that diet or supplements were evaluated as a treatment for GDM, not as a primary prevention. We identified a total of 35 relevant articles (Fig. 1. Legend; Flow-chart of prospective cohort studies and clinical trials included in the systematic review. The search used the following combinations of terms: “diet” or “nutrition” and “gestational diabetes mellitus”. A second search was done with the terms “supplement” and “gestational diabetes mellitus”. Filters; Human; languages: English, French and Spanish; date: up to June 30, 2016). Twenty-eight were related to diet and primary prevention of GDM, eight were clinical trials (Additional file 1: Table S1) and 20 were prospective cohort studies (Additional file 2: Table S2). There were eight articles related to supplements and primary prevention of GDM, six clinical trials (Additional file 3: Table S3) and two prospective cohort studies (Additional file 4: Table S4). Lastly, there was one article [8] that studied whether dietary and supplemental iron were related to the occurrence of GDM (Additional file 2: Tables S2 and Additional file 4: Table S4).

### Quality of the included studies

Of the 14 clinical trials included, only one was not randomized [9]. All women were pregnant when intervention had begun in the clinical trials. The diagnosis of GDM was based on the Oral Glucose Tolerance Test (OGTT), although the measurements of the OGTT was not the same in all articles, some considered the fasting glucose level abnormal when ≥ 5.3 mmol/l [9–11], but others consider it to be abnormal when ≥ 4.8 mmol/l [12], ≥5.1 mmol/l [13, 14] or ≥5.5 mmol/l [15]. Furthermore, the glucose level readings at 1 h and at 2 h were also different. One article studied GDM incidence as a secondary outcome [16] and another one does not give a definition of GDM, although they studied glucose blood levels during the pregnancy of the participants [17].

The observational studies were adjusted for the most important variables (such as age, parity, tobacco products, BMI, physical activity, family history of diabetes, alcohol, ethnicity, total calories consumed). Twelve of these articles [8, 18–28] were based on the Nurses’ Health Study II cohort, which includes more than 13,000 women who reported a singleton pregnancy. In this cohort, GDM cases were identified through self-reported information. The principal measures estimated in these studies were Risk Ratios (RR) and differences in means of GDM incidence.

### Main findings

On one hand, no diet intervention was found to significantly reduce the incidence of GDM except for the intervention of the non-randomized controlled pragmatic trial [11] and the intervention on lifestyle (including diet) of Koivusalo et al. [10], (see Additional file 1: Table S1). Wolff et al. [17] found that an intensive intervention reduces the deterioration of glucose metabolism in obese pregnant women, though they did not report any incidence of GDM. Only two supplement interventions with probiotics and myo-inositol during pregnancy showed a decrease in the rates of GDM compared with a placebo. Luoto et al. [12] intervention showed that probiotics (Lactobacillus Rhamnosus GG and Bifidobacterium Lactis Bb12) reduced the incidence of GDM; 13% (diet/probiotics) versus 36% (diet/placebo) and 34% (control), *p* = 0.003. Luoto et al. [12] explain in their article that probiotic consumption may protect against GDM because these microorganisms can modify intestinal microbiota, altering the fermentation of dietary polysaccharides and improving intestinal barrier function. They also mentioned the importance of the capability of probiotics to regulate the inflammatory pathways. It is understandable that the protection against GDM provided by probiotics, could be mediated through immunomodulatory pathways and polysaccharide fermentation. Moreover, myo-inositol supplements were found to reduce the incidence of GDM in pregnant women (Matarrelli et al. [14]: RR = 0.127; 95% CI, 0.032–0.502; *p* = 0.001 and D’Anna et al. [13]: OR = 0.34; 95% CI, 0.17–0.68; *p* = 0.001) and appear to be an insulin sensitizer. It was reported to reduce plasma glucose levels in insulin resistant conditions such as polycystic ovary syndrome and during the third trimester of GDM pregnancies [29]. The subjacent mechanism of myo-inositol on metabolic benefits is not defined at the moment. It may produce an intracellular effect directly on the activation of acetil CoA carboxylase stimulating lipogenesis. Another theory says that it is a precursor of D-chiro-inositol, which contains inositol phosphoglycan in the extracellular matrix of the cells. It has been proposed that the binding of insulin to specific receptors stimulates D-chiro-inositol, facilitating the transport to the inside of the cell [30]. This explains how myo-inositol interacts in the insulin-signaling cascade [31].

On the other hand, the observational studies collected in Table S2 (Additional file 2) showed that achieving a healthier dietary pattern [25], such a Mediterranean dietary pattern, and lowering the intake of foods with high heme iron content, sugar sweetened cola, potatoes, fatty foods and sweets, can reduce the incidence of GDM, especially among the high-risk population and before getting pregnant [8, 21–23, 32–34].

Known evidence indicates that women who develop GDM have altered functions of β-cells and insulin resistance, limiting their capacity to cope with the metabolic challenges of pregnancy [35]. Similarly, it is known that iron is a redox-active transitional metal, a strong pro-oxidant which promotes the creation of hydroxyl radicals, increasing oxidative stress. The pancreatic β-cell is particularly sensitive to this type of stress due to weak antioxidant protection [36]. Nevertheless, adherence to healthy diets, such as the Mediterranean one, may reduce GDM risk by minimizing such susceptibilities before pregnancy. Common components of these dietary patterns include fruits and vegetables, relatively small amounts of red and processed meats, and high quality carbohydrates. Fruits and vegetables in particular have many antioxidant properties, in addition to providing fiber and micronutrients such as magnesium and vitamin C. The combination of all these factors may protect against metabolic deterioration counteracting free radicals and improving systemic oxidative stress [37].

### Limitations

It is possible that a publication bias may have occurred as there is a limited amount of publications related to the study of primary intervention of GDM through diet, and absorption of supplements, probiotics and other minerals. In addition, the researchers are not discounting the existence of other articles in databases that were not used for this study.

### Clinical applications

In this systematic review we found that the majority of the interventions done during pregnancy are not effective in preventing GDM, and although there were cases where supplements proved to be beneficial, the evidence is very scarce. However, dietary patterns prior to pregnancy seem to reduce the risk of GDM. Programs encouraging young women to achieve a healthy dietary pattern before getting pregnant seem to be the best way to prevent GDM. Measures adopted during pregnancy seem to be ineffective because they require more time to properly curb the development of GDM, especially among the high-risk population.

### Implications for future research

There is a strong need for additional research on this topic. Although some prospective cohort studies have found an association between some nutrients or dietary patterns and the incidence of GDM, the existence of consistency of the results in other populations should be very desirable to prove causal associations between nutritional factors and the risk of GDM. We did not find any controlled trial that evaluated the intervention in dietary pattern before patients get pregnant and the incidence of GDM. Nevertheless, there is an ongoing European multicenter randomized controlled trial in overweight and obese pregnant women on lifestyle intervention and/or vitamin D supplementation [38]. Researchers have recently published the results of the pilot study finding that a healthy eating has a preventive impact on the risk of GDM [39]. Final results of the trial will be published in the near future.

## Conclusions

Overall, since there is evidence that some dietary patterns, such as the Mediterranean diet, seem to lower the risk of developing GDM, and since it has also been shown to prevent other diseases (T2D, metabolic syndrome, cardiovascular diseases), the advice to adhere to this type of dietary pattern could be the best way at present to prevent GDM. Furthermore, it can be a good way to prevent other pregnancy related diseases, such as, hypertension and growth restriction. However, better designed prospective and intervention studies, providing high-quality data are required before conclusions can be drawn about the best intervention for the prevention of GDM.

## Additional files

Additional file 1: Table S1. Characteristics of clinical trials for the primary prevention of Gestational Diabetes Mellitus through dietary factors [9–11, 15–17, 40, 41]. (DOCX 18 kb)
Additional file 2: Table S2. Characteristics of prospective cohort studies for the primary prevention of Gestational Diabetes Mellitus through dietary factors [8, 18–28, 32–34, 42–46]. (DOCX 28 kb)
Additional file 3: Table S3. Characteristics of clinical trials for the primary prevention of Gestational Diabetes Mellitus through supplements [12–14, 47–49]. (DOCX 15 kb)
Additional file 4: Table S4. Characteristics of prospective cohort studies for the primary prevention of Gestational Diabetes Mellitus through supplements [8, 50]. (DOCX 13 kb)

## Abbreviations

GDM: Gestational diabetes mellitus
OGTT: Oral glucose tolerance test
T2D: Type 2 diabetes
