# Supplementary material for: Primary prevention of gestational diabetes mellitus through nutritional factors: a systematic review
Source: BMC Pregnancy Childbirth. 2017 Jan 13;17:30. doi: 10.1186/s12884-016-1205-4 (PMC5237148; doi:10.1186/s12884-016-1205-4)
Supplement: Additional file 1: Table S1. — Characteristics of clinical trials for the primary prevention of Gestational Diabetes Mellitus through dietary factors [9–11, 15–17, 40, 41]. (DOCX 18 kb) [file 12884_2016_1205_MOESM1_ESM.docx]

| **Additional file 1: Table S1.** Characteristics of clinical trials for the primary prevention of Gestational Diabetes Mellitus through dietary factors. | | | | | |
| --- | --- | --- | --- | --- | --- |
| Author, Year, Country | Study design and objective | Participants and sample size | Intervention | Control | Results |
| Asbee et al., 2009, USA.^16^ | RCT  GDM incidence was as a secondary outcome. | Pregnant women  Intervention: n=57,  Control: n=43 | Counseling: Information about gestational weight gain, exercising, diet advice (40% carbohydrate, 30% fat, 30% protein). It started at 6-16 weeks of pregnancy. | Routine prenatal care and standard information booklet on diet and exercise. | No significant difference in the incidence of GDM. |
| Koivusalo SB. et al., 2016, Finland.^10^ | RCT  If GDM can be prevented by lifestyle intervention in pregnant women at high risk of GDM. | High-risk pregnant women at <20 weeks of gestation (history of GDM and/or a prepregnancy  BMI of ≥30 kg/m^2^)  Intervention: n=144, Control: n=125 | Individualized counseling  on diet (optimize consumption of vegetables, fruits and berries, whole-grain products rich in fiber, low-fat dairy products, vegetable fats high in unsaturated fatty acids, fish, and low-fat meat products and a lower intake of sugar-rich foods), physical activity, and weight control. | Standard antenatal care (general information leaflets on diet and physical activity). | The intervention reduced the incidence of GDM (incidence GDM: 13.9% in the intervention group and 21.6% in the control group [95% CI 0.40–  0.98; P = 0.044]). |
| Luoto R. et al., 2011, Finland.^12^ | Cluster-RCT  If GDM can be prevented by lifestyle counseling in pregnant women at high risk of GDM. | 2,271 women were screened by OGTT at 8–12 wk gestation. Euglycemic (n = 399) women with at least one GDM risk factor were included.  Intervention: n=219  Control: n=180 | Individual intensified counseling on physical activity and diet (help to participants achieve a healthy diet containing =/<10% saturated fat, 5%-10% polyunsaturated fat, 25%-30% total fat, and ,10% saccharose of total energy intake, and 25-35 g/d fiber) and weight gain at five antenatal visits. | No counseling beyond usual care, which includes some dietary counseling and follow-up of gestational weight, but only little physical activity counseling. | The intervention failed to have an effect on the incidence of maternal GDM (intervention group 27.3% versus control group 33.0%, p=0.43). |
| Markovic TP. et al., 2016, Australia.^15^ | RCT  To compare the effect of a low–glycemic index versus a conventional high-fiber diet on pregnancy outcomes (including GDM incidence) | 139 pregnant women between 12 and 20 weeks and at high risk of GDM.  Intervention: n=72  Control: n=67 | To follow a low-glycemic index diet (target glycemic index ≤50). | To follow a high-fiber, moderate-glycemic diet, similar to the Australian population average (target glycemic index 60). | No significant differences in GDM incidence (intervention group 13.9% versus control group 13.4%; p=0.917) |
| Mustila T. et al., 2013, Finland.^11^ | Non-randomized controlled pragmatic trial.  The effects of antenatal dietary and physical activity counseling. | Mothers (n = 185) at risk of developing GDM.  Intervention: n=96  Control: n=89 | Between 10–17 gestational weeks. Counselling on diet and physical activity by a public health nurse, and two group counselling sessions by a dietician and a physiotherapist. | The control group’s measures during pregnancy were entered in the questionnaires by the mothers themselves 1–12 months after the end of pregnancy. | The intervention reduced the incidence of GDM. Intervention group 14.6% (8.9% to 23.0%) and control group 29.2% (20.8% to 39.4%), p=0.016. |
| Thornton Y, et al.,  2009, USA.^40^ | Randomized, parallel-group trial.  To compare perinatal outcomes in the control vs the study groups, in adherent and nonadherent patients in the study group, and in those who gain >10 lb with those who gain<10lbs. | Pregnant women with a single fetus between 12 and 28 weeks of gestation and a BMI greater than or equal to 30 kg/m².  Randomized to study group (n=116) and control group (n=116). | Prescribed a balanced nutritional regimen and were asked to record in a diary all of the foods eaten during each day. | Conventional prenatal dietary management. | No statistical differences between study group (9.5%) and control (16.4%) in the incidence of GDM, p=0.118.  Instead, there was statistical differences between adherent group (2.2%) and non-adherent group (34.6%), p<0.01 for incidence of GDM and in those who gained <15lbs (4%) compare to those who gained>15lbs (19%), p<0.01. |
| Vinter CA, et al., 2011, Denmark.^41^ | RCT  To study the effects of lifestyle intervention on gestational weight gain and obstetric outcomes including GDM. | 304 obese women allocated in early pregnancy to lifestyle intervention or control.  Intervention: n=150  Control: n=154 | Dietary guidance, free membership in ﬁtness centers, physical training, and personal coaching. | Received the same initial information about the purpose and content of the study, including access to a website with advice about dietary habits and physical activities in pregnancy, but no additional intervention. | No statistical differences were seen for the incidence of GDM between study group (6%) and control group (5.2%), p=0.76. |
| Wolff S, et al.,  2008, Denmark.^17^ | RCT  10-h dietary consultations can restrict gestational weight gain in obese women and whether this restriction impacts the pregnancy-induced changes in glucose metabolism. | 50 nondiabetic nonsmoking Caucasian obese pregnant women were randomized into intervention group (n=23) or control group (n=27). | 10 consultations 1 hour each with a trained dietitian during the pregnancy. A healthy diet according to the official Danish dietary recommendations. The energy intake was restricted based on individually estimated energetic cost of fetal growth. | No consultations with dietitian and no energy intake or gestational weight gain restrictions. | GDM (Incidence: Not reported)  Restriction of gestational weight gain in obese women is achievable (a gain of 6.6kg in the intervention group vs a gain of 13.3kg in the control group. P=0.002, 95% CI: 2.6-10.8kg) and reduces the deterioration in the glucose metabolism (At 36 weeks of gestation, the s-insulin was further reduced by 23%, -25 pmol/l (-47 to -4, P=0.022) and the fasting b-glucose were reduced by 8% compared with the control group (-0.3 mmol/l, -0.6 to 0.0, P=0.03). |
| *RCT* randomized controlled trial; *BMI* body mass index; *OGTT* oral glucose tolerance test; *CI* confidence interval | | | | | |
