# Supplementary material for: Primary prevention of gestational diabetes mellitus through nutritional factors: a systematic review
Source: BMC Pregnancy Childbirth. 2017 Jan 13;17:30. doi: 10.1186/s12884-016-1205-4 (PMC5237148; doi:10.1186/s12884-016-1205-4)
Supplement: Additional file 2: Table S2. — Characteristics of prospective cohort studies for the primary prevention of Gestational Diabetes Mellitus through dietary factors [8, 18–28, 32–34, 42–46]. (DOCX 28 kb) [file 12884_2016_1205_MOESM2_ESM.docx]

| **Additional file 2: Table S2.** Characteristics of prospective cohort studies for the primary prevention of Gestational Diabetes Mellitus through dietary factors. | | | | | |
| --- | --- | --- | --- | --- | --- |
| **Author, Year** | **Study design and methods** | **Objective** | **Results** | **Covariates** | **Authors’ Conclusions** |
| Bao W. et al., 2013.^18^ | 21,457 singleton pregnancies reported among 15,294 participants of the Nurses’ Health Study II cohort between 1991 and 2001. Generalized estimating equations were used to estimate the relative risks (RRs) and 95% CIs. | To examine the association between dietary protein intake, major dietary protein sources and the risk of GDM. | RRs (95% CIs) comparing the highest with lowest quintiles were 1.49 (1.03-2.17) for animal protein intake and 0.69 (0.50-0.97) for vegetable protein intake. The substitution of 5% energy from vegetable protein for animal protein was associated with a 51% lower risk of GDM (0.49 [0.29-0.84]). For major dietary protein sources, multivariable RRs comparing the highest with the lowest quintiles were 2.05 (1.55-2.73) for total red meat and 0.73 (0.56-0.95) for nuts. | Age, parity, non-dietary and dietary factors, and BMI. Included pregnancies were free of chronic diseases before pregnancy or previous GDM. | *Pre-pregnancy intake of animal protein, in particular red meat, was significantly and positively associated with GDM risk, whereas consumption of vegetable protein, specifically nuts, was inversely associated with the risk. Moreover, substitution of vegetable protein for animal protein, as well as substitution of some healthy protein sources (e.g., nuts, legumes, poultry, and fish) for red meat may potentially lower GDM risk.* |
| Bao W. et al., 2014.^19^ | 21,079 singleton pregnancies from 15,027 women in the Nurses' Health Study II cohort. Since 1991 and every 4 years until 2001, they collected diet information, including consumption of fried foods at home and away from home, using a validated food frequency questionnaire. They documented 847 incident GDM pregnancies. | To prospectively examine the association between pre-pregnancy fried food consumption and risk of incident GDM. | The RRs (95% CIs) of GDM  among women who consumed total fried foods 1-3, 4-6 and ≥7 times/week, compared with those who consumed it less than once/week, were 1.13 (0.97-1.32), 1.31 (1.08-1.59) and 2.18 (1.53-3.09), respectively (p for trend <0.001). The association persisted after further adjustment for BMI (p for trend=0.01). The RR (95% CI) of GDM comparing consumption of ≥4 times/week with less than once/week of fried food consumption away from home was 1.63 (1.15-2.33). | Age, parity, race/ethnicity, family history of diabetes, cigarette smoking, physical activity, total energy intake and diet quality as represented by the Alternate Healthy Eating Index_2010 dietary pattern score. | *Frequent fried food consumption, particularly away from home, was significantly associated with a greater risk of incident GDM.*  *This indicates potential benefits of limiting fried food consumption in the prevention of GDM.* |
| Bao W. et al., 2014.^19^ | 21,411 singleton pregnancies in the Nurses’ Health Study II. 867 incident GDM pregnancies during 10 years follow-up. Pre-pregnancy low-carbohydrate diets (LCD) scores were calculated from validated food-frequency questionnaires. A higher score reflected a higher intake of fat and protein and a lower intake of carbohydrate, and it indicated closer adherence to a low-carbohydrate dietary pattern. RRs and 95% CIs were estimated by using generalized estimating equations with log-binomial models. | To examine the association of 3 pre-pregnancy low-carbohydrate dietary patterns with the risk of GDM. | Multivariable-adjusted RRs (95% CIs) of GDM for comparisons of highest with lowest quartiles were 1.27 (1.06-1.51) for the overall LCD score (p for trend=0.03), 1.36 (1.13-1.64) for the animal LCD score (p for trend=0.003), and 0.84 (0.69-1.03) for the vegetable LCD score (p for trend = 0.08). | Age, parity, race-ethnicity, family history of diabetes, cigarette smoking, alcohol intake, physical activity, total energy intake and BMI. | *Pre-pregnancy dietary pattern relatively low in carbohydrate and high in protein and fat from animal-food sources was positively associated with GDM risk, whereas a dietary pattern relatively low in carbohydrate and high in protein and fat from vegetable-food sources was not associated with the risk.* |
| Bao W. et al., 2016.^20^ | 21,693 singleton pregnancies from 15,632 women in the Nurses’ Health Study II. 854 incident GDM cases during 10 years of follow-up. Pre-pregnancy potato (baked, boiled, mashed or French fries) consumption was assessed from validated food-frequency questionnaires. | If pre-pregnancy potato consumption was associated with the risk of GDM. | RRs of GDM for total potato consumption of 1, 2-4, and ≥5 servings/week, compared with <1serving/week, were 1.20 (0.97-1.48), 1.27 (1.04-1.55), and 1.50 (1.15-1.96), respectively (p for trend = 0.006). | Age, parity, ethnicity, family history of diabetes, smoking, physical activity, total energy intake, alternate healthy eating index 2010 and pre-pregnancy BMI. | *Higher levels of potato consumption before pregnancy are associated with greater risk of GDM.*  *Moreover, substitution of potatoes with other vegetables, legumes, or whole grain foods might lower the risk.* |
| Bowers K. et al., 2011.^8^ | Prospective study. 13,475 women who reported a singleton pregnancy between 1991 and 2001 in the Nurses’ Health Study II. A total of 867 incident GDM cases.  Pooled logistic regression was used to estimate the RR of GDM by quintiles of iron intake controlling for dietary and non-dietary risk factors. | If pre-pregnancy dietary (especially heme iron) iron was associated with the risk of GDM. | RRs (95%CIs) across increasing quintiles of heme iron were 1.0 (reference), 1.11 (0.87–1.43), 1.31 (1.03-1.68), 1.51 (1.17–1.93), and 1.58 (1.21–2.08), respectively (p for linear trend 0.0001). | Age, parity, BMI, physical activity, glycemic load, cereal ﬁber, polyunsaturated fat, current smoking, alcohol, total calories, and family history of diabetes. | *Dietary heme iron intake was positively and significantly associated with GDM risk.*  *No signiﬁcant association was observed between total dietary iron intake and GDM risk.* |
| Bowers K. et al., 2012.^22^ | Prospective study, 13,475 women who reported a singleton pregnancy between 1991 and 2001 in the Nurses’ Health Study II. 860 incident GDM cases.  The adjusted RR of GDM was estimated for quintiles of total fat, specific fat, and the source of fat intakes by pooled logistic regression. | If the total amount and the type and source of pre-pregnancy dietary fats were related to risk of GDM. | Across increasing quintiles of animal fat, RRs (95% CIs): 1.00 (reference), 1.55 (1.20-1.98), 1.43 (1.09-1.88), 1.40 (1.04-1.89), and 1.88 (1.36-2.60) (p for trend = 0.05).  RRs (95% CIs) for dietary cholesterol: 1.00 (reference), 1.08 (0.84-1.32), 1.02 (0.78-1.29), 1.20 (0.93-1.55), and 1.45 (1.11-1.89) (p for trend = 0.04). | Age, parity, current smoking, BMI, physical activity, family history of diabetes, alcohol, race, and total calories. Additional dietary adjustments included cereal ﬁber and mutual adjustment for the speciﬁc fatty acids or source of fats. | *Higher pre-pregnancy intakes of animal fat and cholesterol were associated with elevated GDM risk.* |
| Chen L. et al., 2009.^23^ | Prospective study among 13,475 women who reported at least one singleton pregnancy between 1992 and 2001 in the Nurses’ Health Study II. 860 incident GDM case subjects were identified.  Cox proportional hazards models with multivariate adjustments were applied. | Examined the association between regular sugar-sweetened beverages (SSBs) consumption before pregnancy and the risk of GDM. | Compared with women who consumed 1 serving/month, those who consumed 5 servings/week of sugar-sweetened cola had a 22% greater GDM risk (RR 1.22 [95% CI 1.01-1.47]). | Age, parity, race/ ethnicity, family history of diabetes, smoking, physical activity, alcohol consumption and BMI. | *Pre-pregnancy higher consumption of sugar-sweetened cola (5servings/week) was associated with an elevated GDM risk.* |
| Chen L. et al., 2012.^24^ | Prospective study among 13,475 women with at least one singleton pregnancy in the Nurses’ Health Study II from 1991 to 2001. Dietary intake information was collected by a validated 133-item semi-quantitative food frequency questionnaire designed to assess food intake during the previous year. To determine the consumption of fruit there were nine items and for 100% fruit juices four items. | To examine the association of pre-pregnancy habitual consumption of fruits and fruit juices and the risk of GDM. | The adjusted RRs for GDM from the lowest to highest quintile of whole fruit consumption were 1.00 (reference), 0.80 (95% CI 0.65-0.98), 0.90 (0.73-1.10), 0.80 (0.64-1.00), and 0.93 (0.76–1.16). The corresponding RRs for fruit juice were 1.00, 0.82 (0.66–1.01), 0.78 (0.63–0.96), 0.84 (0.68–1.04), and 1.00 (0.81–1.23). | Age, parity, race, smoking, alcohol intake, physical activity, family history of diabetes, BMI and other dietary factors. | *Pre-pregnancy higher consumption of whole fruits was not associated with increased GDM risk. The association of fruit juices with GDM risk appeared to be nonlinear.* |
| He JR. et al., 2015.^32^ | 3063 pregnant Chinese women from a prospective cohort study. Data on dietary intake were collected using a Food frequency questionnaire at 24-27 weeks of gestation. GDM was diagnosed using a 75 g, 2 h oral glucose tolerance test. Dietary patterns were determined by principal components factor analysis. A log-binomial regression model was used to examine the associations between dietary pattern and the risk of GDM. | To examine the associations between dietary patterns and the risk of GDM in a Chinese population. The analysis identified four dietary patterns: vegetable pattern; protein-rich pattern; prudent pattern; sweets and seafood pattern. | The highest tertile of the vegetable pattern was associated with a decreased risk of GDM (RR 0.79, 95% CI 0.64-0.97), compared with the lowest tertile, whereas the highest tertile of the sweets and seafood pattern was associated with an increased risk of GDM (RR 1.23, 95% CI 1.02-1.49). No significant association was found for either the protein-rich or the prudent pattern. | Maternal age, education level, monthly income, parity, pre-pregnancy BMI and family history of diabetes. | *The vegetable pattern was associated with a decreased risk of GDM, while the sweets and seafood pattern was associated with an increased risk of GDM.* |
| Karamanos B. et al., 2014.^42^ | 1076 pregnant women underwent a 75-g OGTT at the 24th–32nd week of gestation to evaluate for GDM diagnosed by the definition of ADA-2010 and IADPSG_2012. The dietary habits were assessed by a validated questionnaire and a Mediterranean Diet Index (MDI) was computed, reflecting the degree of adherence to the MedDiet pattern of eating. | Explore a possible relationship between the incidence of GDM and the Mediterranean diet (MedDiet) pattern of eating. | -Subjects with GDM had lower MDI (ADA_2010, 5.8 vs 6.3, P=0.028; IADPSG_2012, 5.9 vs 6.4, P<0.001).  -The incidence of GDM was lower in subjects with better adherence to the MedDiet (higher tertile of MDI distribution), 8.0% vs 12.3%, OR=0.618, P=0.030 by ADA_2010 and 24.3% vs 32.8%, OR=0.655, P=0.004 by IADPSG_2012 criteria.  -In subjects without GDM, MDI was negatively correlated with both fasting plasma glucose and AUC glucose, P<0.001 for both. | Age, BMI, family history of diabetes, energy intake and body weight gain during pregnancy. | *Adherence to a MedDiet pattern of eating was associated with lower incidence of GDM and a better degree of glucose tolerance, even in women without GDM.* |
| Qiu C. et al., 2011.^33^ | A prospective cohort study (1996-2008; 3,158 participants) and a case-control study (1998-2002; 185 cases, 411 controls). A food frequency questionnaire was used to assess maternal diet. Multivariable models were used to derive relative risks and 95% CI. | To evaluate if higher egg and cholesterol intakes were associated with increased risk of GDM. | Compared with no egg consumption, adjusted relative risks for GDM were 0.94, 1.01, 1.12, 1.54, and 2.52 for consumption of 1, 2-3, 4-6, 7-9, and >10 eggs/week, respectively (P for trend=0.008) | Total energy, maternal age, race/ ethnicity, pre-pregnancy BMI, physical activity and intakes of meat, ﬁber, vitamin C and saturated fat. | *High egg and cholesterol intakes before and during pregnancy were associated with increased risk of GDM.* |
| Qiu C. et al., 2011.^34^ | A prospective cohort study of 3,158 pregnant women. A food frequency questionnaire was used to assess maternal diet. Multivariable generalized linear regression models were used to derive estimates of RRs and 95% CIs. | To investigate associations of maternal pre-conceptional and early pregnancy heme and non-heme iron intake with subsequent GDM risk. | A 1-mg per day increase in heme iron was associated with a 51% increased GDM risk (RR 1.51 [95% CI 0.99-2.36]).  Non-heme iron was inversely, though not statistically signiﬁcantly, associated with GDM risk, and the corresponding RRs were 1.00, 0.83, 0.62, and 0.61 across quartiles of non-heme iron intake (P for trend = 0.08). | Daily energy intake, age, race/ethnicity, parity, physical activity, pre-pregnancy BMI, dietary ﬁber, vitamin C, saturated fat and cholesterol. | *High levels of dietary heme iron intake during the pre-conceptional and early pregnancy period may be associated with increased GDM risk.* |
| Radesky JS. et al., 2008.^43^ | 1733 women with singleton pregnancies enrolled. Using multinomial logistic regression, they examined associations of first trimester diet, assessed by validated food frequency questionnaire, with results of glucose tolerance testing at 26−28 weeks of gestation. | The aim of this study was to investigate associations of nutrients and foods, including n-3 fatty acids, trans fats, whole grains and dietary patterns, with risk of GDM. | OR (95% CI) for risk of GDM for total dietary fat was 1.00 (0.96-1.05), for saturated fat 0.98 (0.88-1.08), for polyunsaturated fat 1.09 (0.94-1.26), for trans-fat 0.87 (0.51-1.49), and for carbohydrates 1.00 (0.96-1.03) per each 1% of total energy. | Age, pre-pregnancy BMI, race/ethnicity, family history of DM, history of GDM in a prior pregnancy and smoking | *Nutrient or food intake in early pregnancy was not linked to risk of GDM. Nutritional status entering pregnancy, as reflected by pre-pregnancy BMI, was probably more important than pregnancy diet in the development of GDM.* |
| Saldana T. et al., 2004.^44^ | Dietary intake during the second trimester was assessed with a food-frequency questionnaire of 1698 women. Women were classified into 1 of 3 glucose categories: GDM, impaired glucose tolerance (IGT), and normal glucose tolerance. | Examined the relation between macronutrient intake early in pregnancy and the development of glucose intolerance. | Adding 100 kcal from carbohydrates to the diet was associated with a 12% decrease in risk of IGT and a 9% decrease in risk of GDM.  The substitution model showed that substituting fat for carbohydrates (per each 1% of total calories) resulted in a significant increase in risk of both IGT and GDM [RR 1.1 (95% CI: 1.02-1.12) and 1.1 (1.02-1.10), respectively]. | Pre-pregnancy BMI, maternal age, weight gain, physical activity, height and race. | *This study found an association between increased fat intake and the development of glucose abnormalities in pregnancy.* |
| Tobias DK, et al., 2012.^25^ | 21,376 singleton live births reported from 15,254 participants of the Nurses’-Health Study II cohort between 1991 and 2001.  Pre-pregnancy dietary pattern adherence scores were computed based on participants usual intake of the patterns components, assessed with a validated food-frequency questionnaire. | To assess usual pre-pregnancy adherence to well-known dietary patterns and GDM risk. | -aMED (alternate Mediterranean) was associated with a 24% lower risk (RR: 0.76; 95% CI: 0.60-0.95; p for trend = 0.004).  -DASH (dietary approaches to Stop Hypertension) with a 34% lower risk (RR: 0.66; 95% CI: 0.53-0.82; p for trend = 0.0005).  -aHEI (alternate Healthy Eating Index) with a 46% lower risk (RR: 0.54; 95% CI: 0.43-0.68; p for trend, 0.0001). | BMI, physical activity, sedentary time at home, gravidity, smoking status, parental history of type 2 diabetes, and race or ethnicity. The DASH score model additionally adjusted for alcohol. | *Pre-pregnancy adherence to healthy dietary patterns was signiﬁcantly associated with a lower risk of GDM.* |
| Wang Y, et al.,  2000.^45^ | Nulliparous pregnant Chinese women diagnosed GDM (n=56) were compared to groups with normal glucose tolerance (n=77) and glucose intolerance (IGT) (n=38) between 24 and 28 weeks of pregnancy. A 24h recall dietary assessment was also obtained at the time of screening. | To investigate relationships between dietary macronutrient intakes and glucose tolerance in pregnancy. | There was a highly significant reduction in polyunsaturated fat intake in the IGT and GDM groups. | Age, gestational age, height and parity. | *Increased polyunsaturated fat intake was associated with a reduced incidence of glucose intolerance during pregnancy.* |
| Zhang C, et al.,  2004.^46^ | Prospective cohort study (n=755) of pregnant women. The association of maternal plasma ascorbic acid concentrations, measured at an average of 13 weeks of gestation, with subsequent risk of gestational diabetes. Dietary vitamin C intake during the periconceptional period and early pregnancy was ascertained using a semiquantitative food frequency questionnaire. | To assess if vit C could reduce the likelihood of developing GDM. | Plasma ascorbic acid concentrations were inversely associated with the risk of gestational diabetes (p for trend=0.023).  Women with plasma ascorbic acid <55.9 mol/L (lowest quartile) experienced a 3.1-fold increased risk of gestational diabetes (95% CI 1.0-9.7) compared with women whose concentrations were >74.6 mol/L (upper quartile). Women who consumed <70 mg vitamin C daily experienced a 1.8-fold increased risk of gestational diabetes compared with women who consumed higher amounts (95% CI 0.8-4.4). | Age, race, pre-pregnancy adiposity, parity, family history of type 2 diabetes, and household income. | *Diets rich in antioxidants, including vitamin C, could reduce the occurrence of gestational diabetes mellitus.* |
| Zhang C, et al.,  2006.^26^ | 13,110 women who were free of cardiovascular disease, cancer, type 2 diabetes and history of GDM. Subjects completed a validated semi-quantitative food frequency questionnaire in 1991, and reported at least one singleton pregnancy between 1992 and 1998 in the Nurses’ Health Study II. Two major dietary patterns (i.e. “prudent” and “Western”) were identified through factor analysis. | To prospectively examine whether dietary patterns are related to risk of GDM. | 758 incident cases of GDM.  The RR of GDM, comparing the highest with the lowest quintile of the Western pattern scores, was 1.63 (95% CI 1.20-2.21; p for trend=0.001).  The RR comparing the lowest with the highest quintile of the prudent pattern scores was 1.39 (95% CI 1.08-1.80; p for trend=0.018).  The RR for each increment of one serving/ day was 1.61 (95% CI 1.25-2.07) for red meat and 1.64 (95% CI 1.13-2.38) for processed meat. | Age, multiparity, BMI, smoking status, race/ethnicity, family history of diabetes, physical activity, and dietary variables including total fat, cereal fiber, alcohol consumption, glycemic load and total energy intake. | *Pre-pregnancy dietary patterns may affect women´s risk of developing GDM. A diet high in red and processed meat was associated with a significantly elevated risk of GDM.* |
| Zhang C, et al.,  2006.^27^ | 13,110 eligible women in the Nurses´ Health Study II. GDM was self-reported and validated by medical record review in a subsample. | To examine whether pre-gravid dietary ﬁber consumptions from cereal, fruit and vegetable sources and dietary glycemic load were related to GDM risk. | Each 10-g/day increment in total ﬁber intake was associated with 26% (95% CI 9–49) reduction in risk.  Dietary glycemic load was positively related to GDM risk. Multivariate RR for highest versus lowest quintiles was 1.61 (1.02-2.53) (p for trend 0.03). The combination of high-glycemic load and low-cereal ﬁber diet was associated with 2.15-fold (1.04-4.29) increased risk compared with the reciprocal diet. | Age, race/ ethnicity, family history of diabetes, smoking status, BMI, physical activity, intakes of total calories, alcohol consumption, protein and fatty acids. | *Diet with low ﬁber and high glycemic load was associated with an increased risk of GDM.* |
| Zhang C, et al.,  2014.^28^ | Prospective cohort study set in the Nurses´ Health Study II. 20136 singleton live births in 14437 women. 10 years of follow-up. 823 pregnancies affected by incident GDM. | To quantify the association between a combination of healthy lifestyle factors before pregnancy (healthy bodyweight, healthy diet, regular exercise, and not smoking) and the risk of GDM. | The combination of three low risk factors (non-smoker, ≥150 minutes/week of moderate to vigorous physical activity,and healthy eating (top two fifths of Alternate Healthy Eating Index-2010 adherence score)) was associated with a 41% lower risk of GDM compared with all other pregnancies (RR 0.59, 95%CI 0.48-0.71).  The RR (95%CI) for Alternate Healthy Eating Index-2010 diet score in upper two fifths was 0.81 (0.70-0.94). | Age, parity, race/ethnicity, family history of diabetes, history of infertility, year of pregnancy, total energy intake before pregnancy and alcohol intake before pregnancy. | *Adherence to a low risk lifestyle, including adherence to Alternate Healthy Eating Index, before pregnancy was associated with a low risk of gestational diabetes and could be an effective strategy for the prevention of gestational diabetes.* |
| *RR* relative risk; *OR* odds ratio; *ADA_2010* American Diabetes Association_2010; *IADPSG_2012* International Association of the Diabetes and Pregnancy Study Group_2012; *MedDiet* Mediterranean Diet; *AUC* area under the curve | | | | | |
