# Supplementary material for: Primary prevention of gestational diabetes mellitus through nutritional factors: a systematic review
Source: BMC Pregnancy Childbirth. 2017 Jan 13;17:30. doi: 10.1186/s12884-016-1205-4 (PMC5237148; doi:10.1186/s12884-016-1205-4)
Supplement: Additional file 3: Table S3. — Characteristics of clinical trials for the primary prevention of Gestational Diabetes Mellitus through supplements [12–14, 47–49]. (DOCX 15 kb) [file 12884_2016_1205_MOESM3_ESM.docx]

| **Additional file 3: Table S3.** Characteristics of clinical trials for the primary prevention of Gestational Diabetes Mellitus through supplements. | | | | | |
| --- | --- | --- | --- | --- | --- |
| **Author, Year, Country** | **Study design, supplement and objective** | **Participants and sample size** | **Intervention** | **Control** | **Results** |
| D’Anna R, et al., 2015, Italy.^13^ | Randomized, double-center, placebo-controlled trial.  Myo-inositol  To evaluate whether myo-inositol supplementation reduces the rate of GDM and lowers insulin resistance in obese pregnant women. | 220 pregnant women at first trimester, with body mass index 30 or greater, fasting plasma glucose 126 mg/dL or less, random glycemia less than 200 mg/dL and singleton pregnancy were randomized. | Myo-inositol 2 g plus 200 micrograms folic acid twice a day (n=110). | 200 micrograms folic acid twice a day (n=110). | GDM incidence was significantly reduced in the myo-inositol group compared with the control group, 14.0% vs 33.6% (OR=0.34; 95% CI 0.17–0.68; p=0.001). |
| Chan K, et al., 2009, Hong Kong.^47^ | Randomized placebo-controlled trial.  Iron  To test whether iron supplement from early pregnancy would increase the risk of GDM. | 1164 women with singleton pregnancy at less than 16 weeks of gestation with Hb level between 8 and 14 g/dl and no pre-existing diabetes or haemoglobinopathies. | 60 mg of iron supplement daily (n=565). | Placebo (n = 599). | No signiﬁcant difference in the incidence of GDM in the iron supplement and placebo groups at 28 weeks (OR: 1.04, 95% CI: 0.7-1.53; 90% power). |
| Kinnunen T, et al., 2014, Finland.^48^ | Re-analyse data from a RCT of iron supplementation to see whether it supports the risk of GDM found in observational studies. | 2944 less than 16 week pregnant women were randomized at the first visit during their pregnancy. | Routine iron group took elemental iron 100 mg/day throughout the pregnancy regardless of their Hb level (n=1336). | Selective iron group took elemental iron (50 mg twice a day) only if their Hb fell below 100 g/L after 14 weeks gestation on two consecutive visits and anaemia was confirmed in the laboratory (n=1358). | No statistically significant differences in the incidence of the primary outcome [(a composite variable including any glucose intolerance-related outcome (e.g. glucosuria, gestational diabetes, large-for-gestational-age child)] between the selective iron and the routine iron groups (13.0 vs. 11.0%, p=0.12). |
| Luoto R, et al., 2010, Finland.^12^ | Double-blind, placebo-controlled study.  Probiotics  To determine the safety and efﬁcacy of perinatal probiotic-supplemented dietary counseling. | 256 women were randomised at their ﬁrst trimester of pregnancy into a control and a dietary intervention group. The intervention group received intensive dietary counselling provided by a nutritionist and were further randomised, double-blind to receive probiotics or placebo. | -Intervention + probiotics (n=67).  - Intervention + placebo (n=63). | - Control + placebo (n=61). | Probiotic intervention reduced the frequency of GDM; 13% (diet/probiotics) v. 36% (diet/placebo) and 34% (control); P=0.003. |
| Matarrelli B, et al., 2013, Italy.^14^ | Prospective, randomized, double-blind, placebo controlled clinical trial, pilot study.  Myo-inositol  To test whether dietary myo-inositol may improve insulin resistance and the development of GDM in women at high risk. | 75 non-obese singleton pregnant women with an elevated fasting glucose in the first or early second trimester were studied throughout pregnancy. | Supplementation with myo-inositol (n=36) | Placebo (n=39) | The incidence of GDM in mid-pregnancy was significantly reduced (p=0.001) in women randomized to receive myo-inositol compared to placebo (RR=0.13, 95% CI: 0.032–0.502). |
| Zhou SJ, et al., 2012.^49^ | Double-blind, multicenter randomized control trial  n-3 long-chain PUFA (LCPUFA)  To determine whether n-3 LCPUFA supplementation in pregnancy reduces the incidence of GDM. | Pregnant women (n = 2399) of 21 wk gestation were randomly assigned. | DHA-enriched ﬁsh oil (800 mg/d), n=1197. | Vegetable oil capsules without DHA, n= 1202. | No significant difference between intervention and control group was observed (RR=0.97; 95% CI: 0.74-1.27). |
| *Hb* Hemoglobin; *PUFA* polyunsaturated fatty acid; *DHA* docosahexaenoic acid | | | | | |
