# Supplementary material for: Primary prevention of gestational diabetes mellitus through nutritional factors: a systematic review
Source: BMC Pregnancy Childbirth. 2017 Jan 13;17:30. doi: 10.1186/s12884-016-1205-4 (PMC5237148; doi:10.1186/s12884-016-1205-4)
Supplement: Additional file 4: Table S4. — Characteristics of prospective cohort studies for the primary prevention of Gestational Diabetes Mellitus through supplements [8, 50]. (DOCX 13 kb) [file 12884_2016_1205_MOESM4_ESM.docx]

| **Additional file 4: Table S4.** Characteristics of prospective cohort studies for the primary prevention of Gestational Diabetes Mellitus through supplements. | | | | | |
| --- | --- | --- | --- | --- | --- |
| **Author, Year** | **Supplement and objective** | **Study design and methods** | **Covariables** | **Results** | **Authors’ Conclusions** |
| Bowers K. et al., 2011.^8^ | Iron  If pre-pregnancy supplemental iron was associated with the risk of GDM. | Prospective study. 13,475 women who reported a singleton pregnancy between 1991 and 2001 in the Nurses’ Health Study II. A total of 867 incident GDM cases. | Age, parity, BMI, physical activity, glycemic load, cereal ﬁber, polyunsaturated fat, current smoking, alcohol, total calories, and family history of diabetes. | RRs (95%CIs) across increasing quintiles of supplemental iron were 1.0 (reference), 0.98 (0.76-1.25), 0.95 (0.76-1.19), 0.86 (0.68-1.10), and 1.04 (0.84-1.28), respectively (P for linear trend 0.97). | *No significant association was observed between supplemental iron intake and GDM risk.* |
| Helin A, et al., 2012.^50^ | Iron  To investigate the possible association between total daily iron intake (food+supplements) during pregnancy, hemoglobin in early pregnancy and the risk GDM in women at increased risk of GDM. | Prospective cohort study. 399 Pregnant women who were at increased risk of GDM.  Data on iron intake was collected using a 181-item food frequency questionnaire and separate questions for supplement use at 26-28 weeks of gestation. | BMI, age, diabetes in first-degree or second-degree relatives, GDM or macrosomia in earlier pregnancy, total energy intake, dietary fiber, saturated fatty acids and total gestational weight gain. | GDM was diagnosed in 72 women (18.1%).  -Women in the highest quintile of total daily iron intake had an adjusted OR of 1.66 (95% CI 0.84 to 3.30; p=0.15) for GDM.  After excluding participants with low hemoglobin levels (≤120 g/l) in early pregnancy the adjusted OR was 2.35 (95% CI 1.13 to 4.92; p=0.023). | *High iron intake during pregnancy increases the risk of GDM especially in women who are not anemic in early pregnancy and who are at increased risk of GDM.* |
